# Insulin Receptor Substrate-1 Expression Is Increased in Circulating Leukocytes of Patients with Acute Coronary Syndrome

**DOI:** 10.5402/2011/740585

**Published:** 2011-06-16

**Authors:** Manuel F. Jiménez-Navarro, Héctor Bueno, Luis Alvarez-Sala, Noela Rodríguez-Losada, Vicente Andrés, Herminia González-Navarro

**Affiliations:** ^1^Department of Cardiology, Hospital Clínico Universitario Virgen de la Victoria, 29010 Málaga, Spain; ^2^Department of Cardiology, Hospital General Universitario Gregorio Marañón, 28007 Madrid, Spain; ^3^Department of Internal Medicine, Hospital General Universitario Gregorio Marañón, 28007 Madrid, Spain; ^4^Department of Epidemiology, Atherothrombosis and Imaging, Spanish National Cardiovascular Research Center (CNIC), 28029 Madrid, Spain; ^5^Instituto de Biomedicina de Valencia (IBV), Spanish Council for Scientific Research (CSIC) and Fundación Investigación Hospital Clínico de Valencia, Av. Blasco Ibáñez, 46010 Valencia, Spain

## Abstract

The mechanisms underlying the increased risk of cardiovascular disease associated with diabetes mellitus (DM) are not fully defined. Insulin resistance in human metabolic syndrome patients is associated with decreased expression of the insulin receptor substrate-2- (Irs2-) AKT2 axis in mononuclear leukocytes (MLs). Moreover, acute coronary syndrome (ACS) has been linked through genome-wide association studies to the 2q36-q37.3 locus, which contains the *Irs1* gene. Here, we investigated the expression of insulin-signaling pathway genes in MLs from patients with DM, ACS, and ACS plus DM. Quantitative real-time PCR expression studies showed no differences in the mRNA levels of *Irs2, Akt2,* and *Akt1* among all patients. However, *Irs1* mRNA expression was significantly increased in patients with ACS—diabetics and nondiabetics—compared with diabetic patients without ACS (*P* < .02 and *P* < .005, resp.). The present study reveals for the first time an association between increased *Irs1* mRNA levels in MLs of patients with ACS which is not related to DM.

## 1. Introduction

The risk of cardiovascular disease (CVD) and atherosclerosis is 2–5 times higher in patients with diabetes mellitus (DM). Indeed, most DM patients die from CVD complications, mainly myocardial infarction and stroke [1–3]. The prevalence of DM is expected to increase significantly in the next 40 years as a consequence of population aging and the adoption of sedentary lifestyle patterns and is thus set to become a social and health care burden [4]. It is therefore important to understand the molecular mechanisms underlying the acceleration of CVD in DM, in order to identify novel markers and develop preventive and therapeutic strategies.

Insulin resistance (IR) in patients with metabolic syndrome (MetS), who are at high cardiovascular risk, is associated with impaired insulin signaling via the insulin receptor substrate-2- (Irs2-) AKT2 pathway in mononuclear leukocytes (MLs) [5]. In mice, deficiency for the *Irs2* gene produces MetS [6–8], and partial or total inactivation of *Irs2* aggravates atheroma development in the apolipoprotein E-null (apoE^−/−^) mouse model of atherosclerosis [5, 9, 10]. Consistent with the human studies, partial ablation of *Irs2* in apoE^−/−^ mice also reduces IRS2/AKT2 and Ras/ERK1/2-dependent signaling [5], suggesting a causal link between decreased insulin signaling and accelerated atherosclerosis. 

Genome-wide studies suggest a link between acute coronary syndrome (ACS) and the 2q36-q37.3 locus, which encompasses the *insulin receptor substrate-1* (*Irs1*) gene [11]. Here, we analyzed the expression of *Irs2*, *Irs1*, *Akt1*, and* Akt2* genes in MLs from patients with DM (ACS−DM+), ACS (ACS+DM−), and ACS plus DM (ACS+DM+).

## 2. Materials and Methods

### 2.1. Study Population

 Patients aged 40 to 85 years old admitted to Hospital General Universitario Gregorio Marañon (Madrid, Spain) and Hospital Universitario Virgen de la Victoria (Málaga, Spain) between 2007 and 2008 were studied. The cases (DM+ACS+) and ACS controls (DM−ACS+) were patients admitted to the departments of cardiology of the participant hospitals for an ACS. Non-ACS diabetic controls (DM+ACS−) were obtained from patients admitted to internal medicine departments or visited outpatient offices for non-CVD-related causes.

The patients were included in a prospective, hospital-based, case-control study to compare patient differences in the genetic expression of *IRS-AKT*-dependent signaling. DM patients with ACS (ACS+DM+) were compared with age- and sex-matched patients with DM hospitalized for a non-CVD, who had neither prior known CVD nor evidence of CVD according to the physician interview and physical examination plus no evidence of abnormal electrocardiogram and/or ventricular dysfunction on echocardiogram (ACS−DM+) or with ACS without DM (ACS+DM−). DM was defined as fasting plasma glucose >126 mg/dL or previously treated with insulin and/or antidiabetic drugs. ACS was defined as the clinical condition with a written diagnosis in the clinical record of ACS, unstable angina, or acute myocardial infarction, which prompted urgent hospitalization, and was characterized by the following criteria: (1) chest pain or symptom compatible with myocardial ischemia associated with at least one of the following: (a) rise in biomarkers of myocardial necrosis, (b) acute repolarization changes in the ECG, or (c) angiographic evidence of coronary artery disease and (2) absence of extreme pathophysiological condition as a trigger, such as (a) tachycardia of any cause >120 beats per minute, (b) severe hypotension < 80 mmHg, (c) hypertensive emergency (systolic blood pressure > 200 mmHg), and (d) severe anaemia (haemoglobin < 8 gr/dL). The study was approved by the local ethical committees in the two hospitals, and patients were enrolled after providing written informed consent. 

### 2.2. Gene Expression Analysis by Quantitative Real-Time PCR (qPCR)

Total RNA from human MLs was isolated from 3 mL of frozen blood by addition of 7.5 mL *RNAlater *(Ambion) and extraction with the *RiboPure-Blood kit *(Ambion, AM1928). RNA purity and concentration were determined from the A_260/280_ ratio. 0.5–1 *μ*g RNA was reverse-transcribed using the Superscript III First Strand Synthesis Supermix (Invitrogen), and cDNA was PCR amplified with the Platinum Quantitative PCR Supermix-UDG kit and ROX dye (Invitrogen). qPCR reactions were run on a 7500 Fast System thermal cycler (Applied Biosystems), and results were analyzed with the software provided by the manufacturer. The following primers (Forward: Fw; Reverse: Rv) were designed with the Primer Express program (Applied Biosystems): *Irs1*: Fw-5′-CGGAGAGCGATGGCTTCTC-3′; Rv 5′-GTTTGTGCATGCTCTTGGGTTT-3′; *Irs2*: Fw-5′-CCGACGCCAAGCACAAGTA-3′; Rv 5′-GGCCACGGCGAAGTA-3′; *Akt1*: Fw 5′-CCGACGCCAAGCACAAGTA-3′; Rv 5′-CGGCCACGGCGAAGTA-3′; *Akt2*: Fw 5′-CAAGGATGAAGTCGCTCACACA-3′; Rv 5′-GAACGGGTGCCTGGTGTTC-3′; *Gapdh*: Fw 5′-ACCACAGTCCATGCCATCAC-3′; Rv 5′-TCCACCACCCTGTTGCTGTA-3′.

### 2.3. Statistical Analysis

qPCR data were analyzed by one-way ANOVA. Differences in demographic and clinical characteristics were analyzed by one-way ANOVA for quantitative parameters and Chi-square test for qualitative variants, using the SPSS program (Statistical Package for Social Sciences) version 13.0 for Windows (SPSS Inc., Chicago, IU, USA). Outliers were identified by Grubb's test and eliminated. Quantitative variables appear as means ± standard deviation and qualitative variables as percentages. Statistical significance was assigned at *P* < .05.

## 3. Results

The study groups consisted of 35 cases (ACS+DM+), 45 nondiabetic ACS controls (ACS+DM−), and 31 diabetic controls without CVD (ACS−DM+).  Table 1 shows the baseline characteristics according to the study groups. 

Dyslipidemia was more frequent in ACS+DM+ than in ACS−DM+ and ACS+DM− patients (*P* < .007). No differences were detected in arterial hypertension, age, sex, body weight, or smoking among the three groups. 

**Figure 1:**
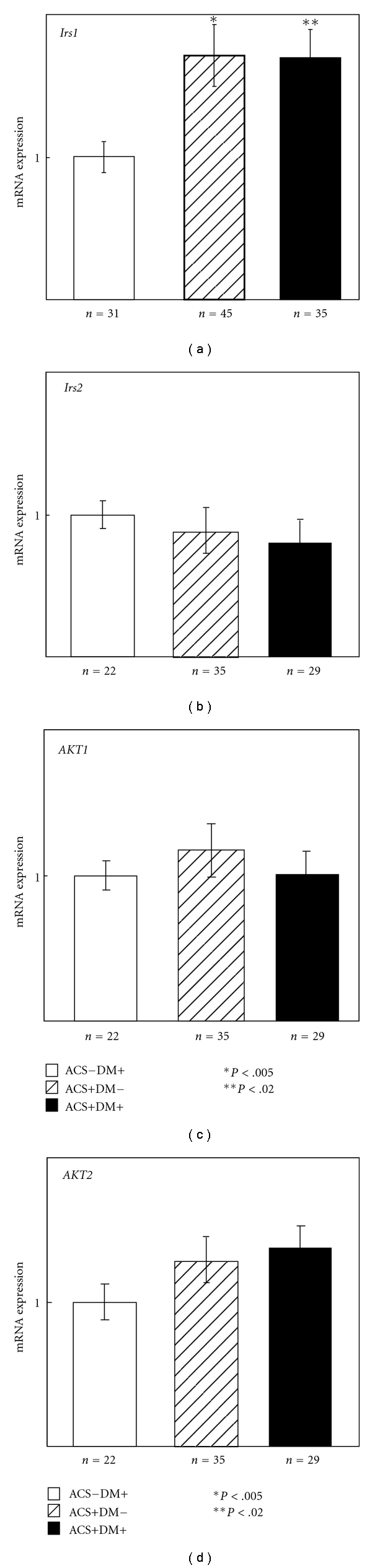
mRNA expression of insulin signaling genes in MLs from patients with ACS+DM−, ACS+DM+, and ACS−DM+. Expression of *Irs1*, *Irs2*, *Akt1*, and *Akt2 *in human WMBCs was analyzed by qPCR. mRNA amounts were normalized to the expression of *Gapdh*, which did not vary between groups, and expression levels are presented relative to the values in patients with DM without ACS (=1).

Previous studies showed that patients with type 2 DM plus IR or IR alone have reduced expression of *insulin receptor* (*Ins-r*)*, Irs2* and *Akt2* in pancreatic *β*-cells, and MLs [5, 12]. Total RNA was prepared from MLs from patients with ACS−DM+, ACS+DM−, or ACS+DM+, and the expression of insulin signaling genes was analyzed by qPCR. The expression of *Akt1*, *Irs2*, and* Akt2* mRNA did not vary between the three patient groups (Figure 1). In contrast, *Irs1* transcript levels were significantly higher in MLs from ACS+DM− (*P* < .005) or ACS+DM+ (*P* < .02) patients than in cells from ACS−DM+ patients. No differences in mRNA expression were observed between ACS+DM− and ACS+DM+ patients, suggesting that increased expression of *Irs1* is associated with ACS independently of DM.

## 4. Discussion

Our results show that *Irs1* mRNA expression in MLs from ACS+DM− and ACS+DM+ patients is higher than in cells from ACS−DM+ patients. Previous studies in our laboratory showed elevated *Irs1 *mRNA expression in MLs from prediabetic IR-MetS patients, who also exhibited increased CVD risk [5]. These findings are consistent with genome-wide studies that suggest linkage between ACS and the 2q36-q37.3 locus, which contains the *Irs1 *gene [11]. More specifically, the IRS1 972Arg variant has also been associated with a 3-year post-ACS mortality [13], suggesting a role of IRS1 in ACS complications. In the present study, *Irs1* mRNA levels in ACS+DM− and ACS+DM+ patients were similar, suggesting that the presence of DM does not have an effect on* Irs1* expression.

Interestingly, myocardial infarction induced by artery ligation in nondiabetic rats causes a partial impairment of insulin response that is associated with cardiac contractile dysfunction [14]. ACS in human patients might produce a similar defect in insulin-mediated signaling, resulting in a compensatory increase in *Irs1* expression. The present findings suggest that *Irs1* expression levels might be a marker of ACS; however, further studies are required to assess the importance of increased *Irs1* expression in ACS and to define the underlying molecular mechanisms.

## 5. Conclusion

In the present study, we demonstrate an association between increased *Irs1* mRNA levels in human ML of patients with ACS which is not related to DM and suggest that *Irs1* levels might be a potential marker for ACS.

##  Conflicts of Interests

The authors declared that there is no conflicts of interests.

##  Authors' Contributions

M. F. Jiménez-Navarro participated in the design, acquisition of human samples, and data and contributed to the writing of paper; H. Bueno participated in the design and acquisition of human samples and contributed to the writing of paper; L. Alvarez-Sala participated in the design of the study and revised critically the paper; N. Rodriguez-Losada participated in the acquisition of human samples and revised critically the paper; V. Andrés participated in the design of the study and wrote the paper; H. González-Navarro participated in the design of the study, performed expression studies and analysis of data, and wrote the paper.

## Figures and Tables

**Table 1:** Baseline characteristics according to study group.

	ACS−DM+	ACS+DM−	ACS+DM+	*P* value
	*n* = 31	*n* = 45	*n* = 35	
Age in years (average ± SEM)	62.7 ± 2.0	65.1 ± 2.5	65.1 ± 1.9	.36
Sex (% male)	68.8	81.1	68.3	.36
Body weight Kg (average ± SEM)	72.8 ± 2.0	80.0 ± 2.9	78.1 ± 2.6	.15
Heigh cm (average ± SEM)	164.8 ± 1.1	165.6 ± 1.7	165.8 ± 1.5	.85
Arterial hypertension (%)	62.5	54.1	73.2	.21
Dyslipidemia (%)	56.3	29.7	63.4	.007
Smoking (%)	57.4	64.9	58.5	.77

## Abbreviations

ACS: Acute coronary syndrome
DM: Diabetes mellitus
IRS: Insulin receptor substrate
MetS: Metabolic syndrome
ML: Mononuclear leukocytes
CVD: Cardiovascular disease
qPCR: Quantitative real-time PCR.
